# Biloma Secondary to Percutaneous Liver Biopsy Case Report

**DOI:** 10.1155/2020/9605370

**Published:** 2020-03-03

**Authors:** Marcos Aranda, Jacqueline Mulhall, Alexander Friedman, Joel Brockmeyer

**Affiliations:** Dwight D Eisenhower Army Medical Center, Fort Gordon, Georgia

## Abstract

Biloma and biliary leak after percutaneous liver biopsy (PLB) are rare. Previous cases are largely in the setting of transplant, oncology, and cirrhotic patients. Patients can be asymptomatic, peritoneal, or present with obstructive symptoms, including bilirubinemia. A 55-year-old male referred for transaminitis attributed to nonalcoholic fatty liver disease (NAFLD) underwent an ultrasound- (US-) guided PLB. He returned the same day with abdominal pain, normal vitals, a nontender abdomen, and a leukocytosis. He was found to have a subcapsular fluid collection attributed to a hematoma. He underwent observation and was discharged. He presented 4 days later with fever, tachycardia, leukocytosis, and bilirubinemia. CT demonstrated growth of the subcapsular fluid collection. Percutaneous drainage revealed bilious fluid. He was transferred for endoscopic retrograde cholangiopancreatography (ERCP). A right biliary branch was stented, and he was discharged the following day with antibiotics. US should be utilized for percutaneous biopsies to avoid biliary complications. Typical presentations of biliary complications include abdominal pain and biliary obstruction. The differential diagnosis for perihepatic and subcapsular fluid collections after PLB should include bile. ERCP should be offered for the treatment of larger or symptomatic collections.

## 1. Introduction

Percutaneous liver biopsy (PLB) is frequently indicated in the workup of many common conditions including nonalcoholic fatty liver disease (NAFLD), and the procedure is completed frequently with few complications [1]. Common complications include pain, superficial bleeding, hematomas, hemobilia, and intraperitoneal hemorrhage [2, 3]. Less common complications include transient bacteremia, biloma, biliary leak, injury to additional organs, such as the right lung or colon, and death [4, 5]. Boyum et al. found an overall complication rate of 0.7% in over 6000 biopsies of which 0.5% were complications related to bleeding [2]. Seeff et al. found only two episodes of biliary complications in over 2500 patients, and both were related to direct injury of the gallbladder [3].

Paymani et al. reported 4 cases of bile leak in transplant patients, one of which was intraparenchymal and three were transhepatic, and they all reported obstructive symptoms [6]. Don et al. reported one case out of 99 PLB in pediatric transplant patients [7]. Avener et al. reported one case in 1979 of an asymptomatic leak diagnosed incidentally after biliary obstruction secondary to lymphoma [8]. Ruben et al. reported one case of bile peritonitis in the setting of uncompensated cirrhosis [9]. Ahluwalia and LaBrecque described the only other published case of a symptomatic biloma in a nontransplant patient without cirrhosis, which resulted in mass effect with gastric outlet obstruction [10].

## 2. Case Presentation

A 55-year-old male was referred to a gastroenterologist for a persistent mild transaminitis of an AST in the 40s and an ALT in the 80s. He had a history of hyperlipidemia, obesity with a BMI of 38, and sleep apnea. He took simvastatin 80 mg daily with no antiplatelets or anticoagulants. He consumed about 3 to 4 alcoholic beverages per week, and he had no family history of cirrhosis. Viral serologies were negative, and iron and copper studies were normal. Serologies for autoimmune hepatitis, primary biliary cirrhosis (PBC), and primary sclerosing cholangitis (PSC) had been normal.

**Figure 1:**
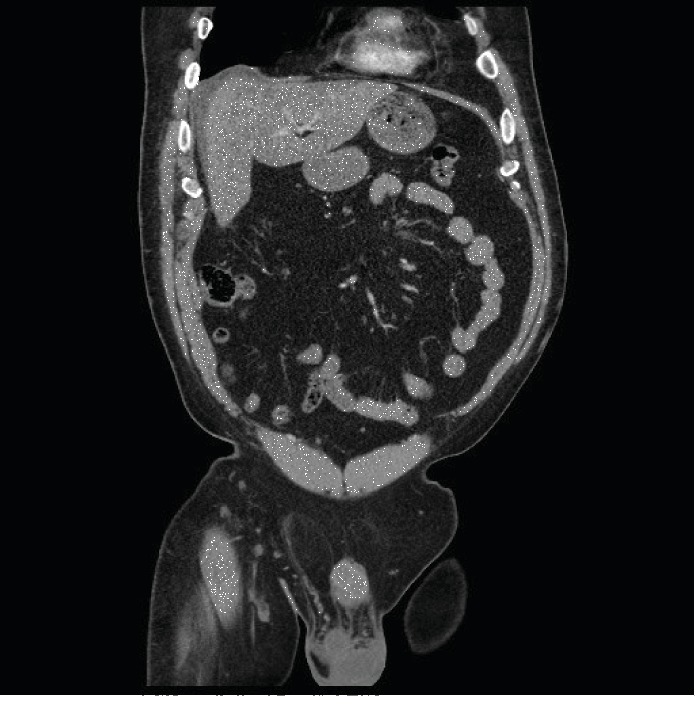
2 cm nonenhancing subcapsular fluid collection coronal.

**Figure 2:**
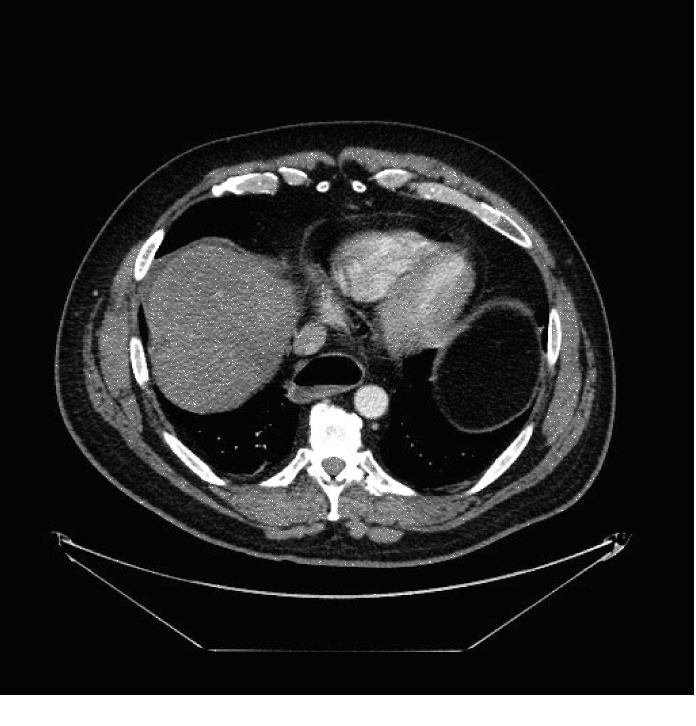
2 cm nonenhancing subcapsular fluid collection axial.

He was referred to a radiologist for an ultrasound- (US-) guided biopsy. His INR was 1.1, and his platelets were 236,000 cells/*μ*l. A 17 G needle was used for anesthesia and a 14 G needle for sampling. After the procedure, he had a collagen thrombin slurry embolization of the tract. Later that day, he presented with right upper quadrant pain, transient tachycardia to the low 100 s, and tenderness in the right upper quadrant without rebound. Studies revealed a leukocytosis of 13,900 cells/*μ*l, a total bilirubin of 1.2 mg/dl, and a CT of the abdomen and pelvis with intravenous (IV) contrast revealed a 2 cm subcapsular fluid collection which was attributed to a hematoma (Figures 1 and 2). He was admitted for 24 hours of observation with improved pain control and was discharged.

**Figure 3:**
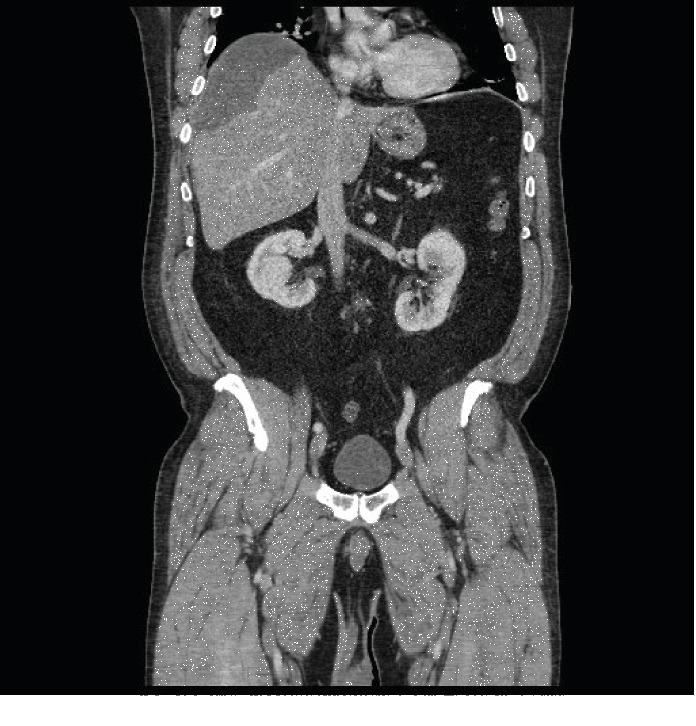
12 cm subcapsular fluid collection coronal.

**Figure 4:**
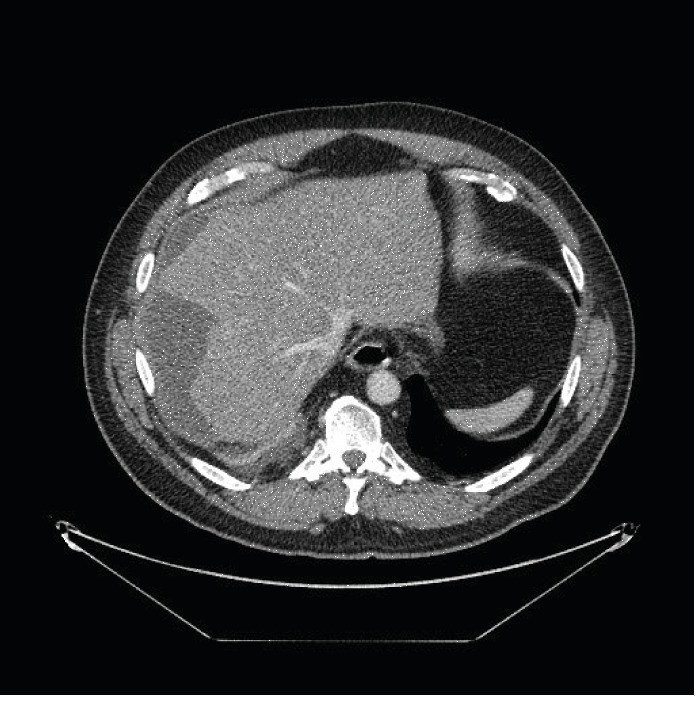
12 cm subcapsular fluid collection axial.

He returned 4 days later with worsening pain, shortness of breath, and loss of appetite. He was febrile to 102.3 F and tachycardic to the 110 s and was tender without peritoneal signs in the right upper quadrant. He had a persistent leukocytosis, and his total bilirubin increased to 3.3 mg/dl. A CT with IV contrast was repeated with enlargement to 12 cm of his subcapsular fluid collection (Figures 3 and 4).

The next day, he underwent percutaneous drainage with return of 20 m of green bilious fluid, and a pigtail catheter was left in place after the diagnosis of biloma was confirmed. The same day he was transferred to a facility with ERCP (endoscopic retrograde cholangiopancreatography) capabilities which demonstrated a bile leak from a small, distal duct of the right lobe which was stented with a 5 cm, 10 F plastic stent of which no images were recorded. He was discharged the following day on a week of amoxicillin/clavulanate.

At a clinic follow-up a week later, his pain had improved, his vitals had normalized, and his drain output had diminished. The drain was removed, and he was arranged a follow-up 6 weeks later for stent removal.

## 3. Discussion

While the initial presentation after biopsy may have represented subcapsular bleeding rather than bile, the bile seen on percutaneous drainage and bile leak on ERCP more strongly suggest an early and clear association between the biopsy and the bile leak. Our case varies from the current literature in that our patient had no history of cirrhosis, malignancy, or transplant. While our patient was symptomatic, he did not have peritoneal signs or signs of obstruction, which is relatively uncommon.

Both gastrointestinal and radiologic societies have developed guidelines regarding the prevention of bleeding, but there are few specifics to the prevention of biliary complications [1, 11, 12]. General guidelines to prevent biliary complications include utilizing US, which can assist in avoiding large bile ducts or the gallbladder [1]. At least a 16 G, 2 cm needle should be utilized to obtain a sufficient pathologic specimen, and small needles should not be used to avoid biliary complications [1]. Once recognized, percutaneous drainage can be performed to diagnose and treat the complication [13–15]. Large or persistent fluid collections require ERCP to diagnose and prevent ongoing leak [13–15]. If a collection is infected, adequate drainage should be assured and antibiotics should be initiated [13, 15].
